# No best, only better: another APSTH milestone

**DOI:** 10.1186/s12959-016-0091-3

**Published:** 2016-10-04

**Authors:** Ming-Ching Shen

**Affiliations:** 1 Department of Internal Medicine, National Taiwan University Hospital, Taipei, 100 Taiwan; 2 Department of Internal Medicine, Changhua Christian Hospital, Changhua, 500 Taiwan

It begins with the *Sino-Japanese Symposium on Coagulation, Fibrinolysis, and Platelet* first held in Hanchow, China in 1990, and again in Taipei, Taiwan in 1992. Thanks to the persistence and hard work of the conference founders and the participation of colleagues from countries around the world, that small, oligo-country conference has evolved into a regional conference now held every two years in cities of the Asia-Pacific region. Since its launch in Taipei, Taiwan in 2000, the APSTH Congress has been, and is now recognized as a key event in our field of thrombosis and hemostasis research.

Taiwan has the honor of hosting the congress for the second time in 16 years with a program designed to foster international exchange and friendship in the pursuit of the newest medical knowledge on thrombosis and hemostasis. The best proof of our persistence in this endeavor is now lying before you, **State of the Art 2016: Research and Review from the 9th Congress of the Asian-Pacific Society on Thrombosis and Hemostasis**, the first-ever publication of our APSTH conference proceedings. Truly, another big milestone.

The contributions in this first edition of APSTH proceedings consist of a balanced mixture of basic and clinical research and review papers. It is our hope that this APSTH publication will provide insight and help to advance our understanding in the various fields of thrombosis and hemostasis. We are grateful to the contributing authors for their excellent work. We are indebted to the many international reviewers who rose to the challenge of publication time constraints to provide us their insights and opinions. We wish to acknowledge the guidance of the editorial office of *Thrombosis Journal*, Dr. Yukio Ozaki, and Rachael Sykes from the supplements team at BioMed Central, and the support of Toby Hung, Jeff Boice, and Shih-Yao Lin which were instrumental and invaluable in the preparation and publication of these proceedings. Thank you all.

We wish you happy reading.

Supplement Editorial Committee, APSTH 2016

Ming-Ching Shen, organizer

Shu-Wha Lin, co-editor

Ching-Ping Tseng, co-editor

Jiaan-Der Wang, co-editor

